# Framing professional programs within development projects: driving longer term recognition and integration

**DOI:** 10.1186/s12909-016-0633-1

**Published:** 2016-04-19

**Authors:** Diane Wallace, James Loughman, Kovin Naidoo

**Affiliations:** University of KwaZulu Natal, Durban, South Africa; Dublin Institute of Technology, Dublin, Ireland; Brien Holden Vision Institute, Durban, South Africa

**Keywords:** Regulation, Optometry, Development

## Abstract

**Background:**

Optometry has, over the past ten years, emerged as a profession strategically positioned to address the burden of uncorrected refractive error in developing countries. Estimates suggest that 285 million people in the world are needlessly visually impaired, mainly due to the lack of trained eye health professionals in the developing world. Development initiatives in eye care have therefore moved away from vertical, service delivery approaches to supporting the establishment of more sustainable, locally owned training programs. This research is based on one the evaluation of one such initiative known as the Mozambique Eyecare Project.

**Methods:**

This study followed a qualitative research design. Ethical approval was granted by the Research Ethics Committee at the Dublin Institute of Technology, which followed the tenets of the Declaration of Helsinki. A qualitative, interview-based study was undertaken between 2012 and 2014 with eighteen key informants involved in the design, planning and implementation of the project. A semi-structured interview guide was developed to explore, inter alia, challenges relating to the establishment of the new profession of optometry in Mozambique. Data was coded and analysed thematically and results derived from a process of descriptive-interpretive analysis.

**Results:**

The establishment of a new profession within the ambit of a development project presents several challenges, principally the establishment of the profession's identity in relation to similar professional cadres' in-country. The risk of not addressing professional regulatory requirements for new programs, where equal or similar qualifications have not previously existed, are that the profession may not be officially recognised by the relevant health authorities and therefore not mainstreamed into public health services, or that training standards and scope of practice may be inappropriate to local needs. Overall, the public may become vulnerable to unscrupulous health care practices.

**Conclusions:**

Health professions are regulated in order to ensure patient safety, as well as minimum standards of care and training within professions. Development projects must address issues of professional identity and official recognition of health professions and their respective qualifications through relevant local authorities, so that graduate qualifications are legitimised and the longer term objectives of the development investment are supported.

## Background

The development of eye care services has found its way into the national plans of most developing countries in response to global advocacy initiatives such as VISION 2020: The Right to Sight. Estimates suggest that 285 million people in the world are needlessly visually impaired [32], mainly due to the lack of access to refractive services in the developing world; a consequence of the dearth of appropriately trained eye health professionals [20]. The vision of the World Health Organization (WHO) is a world in which nobody is needlessly visually impaired and where there is universal access to comprehensive eye care services. World Health Assembly resolution 66 therefore encourages the development and implementation of integrated national eye health policies, plans and programmes to enhance eye health and strengthen health systems [31].

Visual impairment due to uncorrected refractive error (URE) is easily correctable by means of spectacles or contact lenses; yet millions of people in the developing world suffer the unnecessary economic and social consequences associated with URE [10]. Central to this challenge is the need to develop a professional workforce that is adequately trained to manage the burden of URE in developing countries. In 2002, Holden and Resnikoff proposed optometry as a profession centrally positioned to meet this need [11]. Recent initiatives to address the human resource gap have, therefore, included the establishment of Schools of Optometry in countries or regions in need [18].

Mozambique is a country of 25 million people [27] with a huge unmet need for refractive care services, scarce human resources and limited capacity for the delivery of eye health services [16, 18, 26]. In 2009, the Mozambique Eyecare Project (MEP) was established as a regional School of Optometry for Lusophone Africa, funded through the Programme for Strategic Cooperation between Irish Aid and Higher Education and Research Institutes 2007-2011. The principal objective of the MEP was to produce optometric professionals, through a newly established training program at Universidade Lúrio, who could address the eye care needs of an unserved region where optometry as a profession did not previously exist. Addressing a longer term developmental need of human resource capacity development for eye health, the MEP ventured in relatively new territory in response to a growing health systems strengthening approach since within development circles, eye health projects have historically tended more toward service delivery approaches [2]. The involvement of development organisations in the establishment of formal, institutionalised professional optometry training programmes was therefore an emerging concept.

The development of new health care training programs in low income countries has historically been in response to identified need or specific policy positions adopted by governments or global advocacy organisations [12]. Delivering change is, however, a complex and challenging process [23]. The time and effort required to plan for projects on which major development outcomes depend is also often underestimated [9].

This research was designed to explore key factors that impact the establishment of a new healthcare profession in a developing country. Furthermore, the study set out to evaluate the structural considerations necessary in the design, planning and implementation of development-led projects in optometry, which would support shorter term project efficiency, and longer term program sustainability.

Very little research has been conducted to identify and document operational considerations in this regard. Given that the development of Schools of Optometry is progressing in other countries, understanding the real program developmental challenges and specific contextual dynamics, will help to shape planning processes for new optometry training programs, as well as address potential barriers to implementation.

The aim of the study was therefore to evaluate the MEP in order to develop a framework for development-led training projects in optometry. More specifically, the study sought to identify key factors affecting the implementation and longer term sustainability of such initiatives, and make recommendations on possible solutions to mitigate these.

## Methods

This study followed a qualitative research design. Ethical approval was granted under the MEP by the Research Ethics Committee at the Dublin Institute of Technology (DIT) which followed the tenets of the Declaration of Helsinki.

Data was collected by means of key informant interviews and validated by document review methodologies. An interview guide consisting of probe questions was used to facilitate semi-structured interviews. Sampling was purposive (Table 1), with the following eligibility criteria for inclusion in the study:

**Table 1:** Study sample showing Key Informant Interviewee’s designations

Category	Number of Interviewees	Designation/s
Partner 1 (BHVI)	4	Global Programs Director
South Africa		Director for Human Resource Development
		Programs Manager: Africa
		Sub-regional Manager: Southern Africa
Partner 2 (UL)	4	University Rector
		Senior Faculty Member
Mozambique		
		Administrative Director
		Coordinator: Optometry
Partner 3 (DIT)	1	Head: Optometry Department
Ireland		
Partner 4 (UU)	1	Faculty representative: Optometry
Northern Ireland		
Project Team	3	Project/Grant Director (DIT)
		Project Manager
		Project Administrator
Faculty	4	Optometry Lecturing Staff (UL)
Research Student	1	Scholarship recipient

- ✓ Direct implementation of the project at an operational level;
- ✓ Management of the project; or
- ✓ Strategic oversight of the project.

A total of 21 people were identified as key informants. Eligible participants were approached in person or via email, briefed on the research study and the reason for their selection, and respective verbal or written consent to participate was sought. One identified key informant agreed to participate in the research, but did not avail themselves for an interview, and two could not be interviewed due to scheduling and logistical challenges across countries. Therefore, eighteen key informants were ultimately successfully recruited to the study.

Eighteen interviews were conducted with key informants involved in the design and implementation of the project. In-person interviews were scheduled and held in Mozambique, Ireland and South Africa, except for one interview which was conducted via Skype. Verbal consent was sought and data was anonymised. Verbal data was transcribed by the interviewer, and interview notes were reviewed within 48 hours of the interview to translate the shorthand to complete sentences so the richness of the data was not lost.

Interview transcripts were analysed and systematically coded by the researcher, who was also the interviewer. An inductive approach to coding was used, which does not impose a predetermined structure, but rather uses the actual data to derive the structure of the analysis [3]. Inductive analysis involves discovering patterns, themes or categories in one’s data [1]. Data was manually coded and recoded using meaning units, which are parts of the data communicating sufficient pieces of information which provided meaning to the researcher. The development of themes arose from a process of pattern recognition and constant comparison of data from various sources. Emerging themes were identified and organised using thematic analysis [6]. Using document analysis [14], secondary sources of information were examined in relation to key issues or concerns emerging from the literature and primary data.

Documents for review included project reports, minutes of meetings and email correspondence made accessible to the researcher by project personnel in the period 2009-2011. Document content was analysed by the researcher using abductive reasoning, which is a form of logical inference used to find the most likely explanation for observations made herein [22].

## Results

**Fig. 1:**
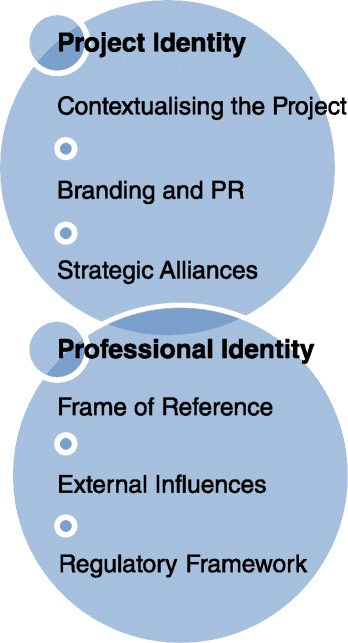
Factors influencing project identity and professional identify within higher education development projects

Two key interrelated “Identity” themes impacted the project and the optometry program, particularly in the early stages of the MEP. These included “Project Identity” and “Professional Identity” which each included several key components as outlined in Fig. 1.

## Project identity

A surprising finding in the research was the reported reservations on the part of some local representatives to the proposed new MEP training initiative. Local officials reported an initial mistrust towards this “ambitious” new training idea as a result of prior institutional experience relating to challenges in the establishment of another new program.

In 2008, following early talks amongst proposed MEP partners, a mass screening ‘Campaign for Quality Vision’ was initiated by Universidade Lúrio to introduce the concept of optometry to Mozambique. This hugely successful event saw more than 3000 people have their vision screened for the first time, and had the former first lady of Mozambique, Graça Machel open the event.

*“One of the main things that propelled the decision to start the optometry course was the experience from the first Campaign for Vision Screening. Graca Machel was in attendance. There was the idea to bring optometrists from all over the world together and expose trainees to the activities they would be involved in. This made the university take the decision to start the program* [sic]*.*- Key Informant 2 (Senior Partner representative, Mozambique)

The success of this screening campaign and the associated publicity therefore ignited local support towards, and enthusiasm around, the MEP and the proposed optometry program.

### Contextualising the project

One of the major issues highlighted by the local partner was that there was an initial lack of distinction between the MEP and the optometry program.

*“The project started one year after the course was launched. Initially it was difficult to see clearly what was the project and what was the optometry course itself. There was a perception that the project was there to support the course, but it was not understood at the time that the project had its own life and activities* [sic].”- Key Informant 2

The MEP represented a five year project within a broader program for optometry development in Mozambique. However, the lack of distinction between the shorter term project and the longer term professional training program created misunderstanding amongst partners.

“*Initially, the implementing people* [local stakeholders] *did not know about the project. They didn’t understand what the funding was for.”*- Key Informant 8 (Project Administrator, Ireland)

Additionally, it was reported that Universidade Lúrio had expectations that the project would be responsible for all activities relating to delivery of the optometry program, though this was not the case.

“*Partners eventually came to a mutual understanding of the project’s activities in relation to the longer term program, and this merger was good for the course*”.– Key Informant 2

The above highlights the importance of clarifying the purpose of development funding, as well as partner roles and responsibilities.

### The value of branding and a public relations strategy in development work

It was reported during interviews that the recognisable MEP brand was significantly valuable in both attracting suitably qualified faculty to the project, and later accessing additional funding to support the longer term sustainability of the program. The factors identified as having a positive influence on the MEP’s identity included:

- the MEP’s brand strategy,
- strategic alliances,
- local political support, and
- emphasis on the MEP, rather than individual partner brands.

Ilal and Kleibl [13] also highlighted the MEP’s strong media presence as key to drawing attention to the project, and the generation of positive publicity for the project.

### Strategic alliances

The Mozambique Eye Care Coalition (MECC) is a coalition of development organisations involved in eye health projects in Mozambique, of which the MEP became a member. Its purpose was to engage in collective advocacy with the Mozambican government for a comprehensive, co-ordinated eye health strategy, in support of the National Plan for Eye Health [17]. Results from interviews and reports indicated that the MECC was a valuable advocacy network and strategic platform through which project partners drove support for the MEP’s activities, and specifically, recognition of optometry as a profession within Mozambique. Furthermore, collaboration with other local and international development and advocacy organisations such as the World Council of Optometry, African Council of Optometry and the International Agency for the Prevention of Blindness also reportedly created opportunities for sharing knowledge and experiences relating to optometry development, further enforcing the project’s identity.

The MEP engaged senior politicians such as the Head of State, consular officials and the First Lady in key activities relating to the project such as institutional visits, official trips to Ireland and graduation ceremonies. It was reported that demonstrating value to, and through, these senior officials promoted acceptance by local communities and drove public support for the project.

However, while there was some engagement with high level government officials at different stages of the project, the results herein suggest that a targeted, consistent and collaborative approach to engaging government was lacking. Much of the earnest engagement from the MEP in relation to official recognition of this new profession of optometry focussed on the Ministry of Health (MoH) initially. Engagement with the Ministry of Education began much later in the project when the resistance to the training of optometrists by some quarters in the MoH was identified.

## Professional identity

Optometry as a profession had not existed in Mozambique prior to the MEP and there was, therefore, a reportedly poor frame of reference to the profession initially and lack of perspective as to how the profession might integrate with the other professions to constitute an eye health team.

### Introducing a new profession: frame of reference

Several informants reported that there was a strong frame of reference to the profession of ophthalmology in Mozambique, and generally a poor understanding of where optometry would fit within the health system. This was underscored by ophthalmology’s central positioning in the National Eye Health plan and the absence of any reference to optometry.

Key informant 2 reported having been exposed to optometry in Brazil and associated their positive response to the MEP with their observation of optometrists “doing a nice job in communities” in that country. While there was initial scepticism towards the optometry training program by some, the usefulness of the optometry course was later acknowledged by local officials due to the unaddressed need for eye care, particularly in the northern provinces of Mozambique, since most of the eye care being delivered in the country was concentrated in and around the capital city of Maputo in the south, delivered primarily by ophthalmologists who are medically trained eye specialists focusing on ocular disease management and surgery, with no refraction or vision correction services which are primarily the domain of optometrists.

### External influences

The misunderstanding of the skills level and scope of practice of optometrists, as well as the perceived overlap in training as compared to ophthalmologists, were cited as some of the reasons for the initial resistance to the training of optometrists in Mozambique within ophthalmological circles. Regional alliances were noted as influencing perceptions in this regard. Senior officials in Mozambique reportedly retained strong links with Brazil which had a shared history of colonisation by Portugal in the early 16^th^ century. Local informants reported during interviews that this association negatively influenced the perceptions around optometry and its initial acceptance, since similar professional antagonism between the professions of optometry and ophthalmology existed in Brazil.

*“We are importing a problem from a different culture, with a different perspective.”* - Key Informant 2

Through the project and its activities, Universidade Lúrio officials reportedly came to appreciate the difference in scope between the professions of optometry and ophthalmology, and expressed recognition that the two professions could work together to help address the eye care needs in Mozambique, and thereby make a significant impact in addressing the burden of visual impairment. However, results suggest that the lack of clarity of the roles of the professions may have impacted on the delays in gaining acceptance in the MoH, as key drivers of the eye health agenda in Mozambique were ophthalmologists, including the National Eyecare Coordinator responsible for informing the development of the National Eyecare Plan for Mozambique.

Further to this, despite technical expertise from project partners at the Brien Holden Vision Institute (BHVI) and DIT into the curriculum design and academic program structure for the MEP, further input was independently sought by the local partner from allies in Brazil, Portugal and Spain, which was not in keeping with the spirit of the partnership, or strategy of the project. The models of training in these countries are not standardised, however they ultimately influenced the final curriculum and model of training for the MEP. It was accepted in the spirit that local sentiments needed to be accommodated for purposes of moving forward with the project. While these influences were significant in terms of the model of training eventually adopted by the MEP, they were insignificant in terms of curricula inclusions towards the World Council of Optometry’s requirements for Level 3 optometry training as was the plan for the MEP.

### Need for a regulatory framework

One local key informant cited the absence of a regulatory framework for optometry in Mozambique as a key challenge in guiding the profession and its integration within the health system locally. The graduate placement strategy for the MEP was that optometrists would be employed within the public health sector and deployed to geographical areas of need. Inherent in this was the requirement that posts would be available for optometrists to be employed within the MoH. Several participants also cited the risk of graduates not being placed within the MoH as one of the biggest threats to the project achieving its objectives. Interviewees cited weaknesses in advocacy efforts on the part of the MEP partners as a contributing factor to posts not having been created by the MoH at the time of graduation for the first cohort of optometrists.

*“Posts were supposed to be created ready for graduates on completion. After the first graduation, posts were not readily available and it took almost one year for graduates to take up employment with the Ministry of Health. This can be highlighted as a gap in stakeholder engagement. Project partners should have followed up on roles and responsibilities, but this was not done well* [sic].*”-* Key Informant 4 (Partner representative, South Africa)

## Discussion

Introducing a new profession to a country warrants several considerations in terms of profiling the scope and role of the profession within the local health system. When professional programs are initiated by means of development-funded projects, further consideration needs to be given to factors which influence the acceptance and understanding of the role of these new professional cadres.

Managing local perceptions, and implementing a wider development agenda which includes the community perspective and the support of government agencies, have been cited as key challenges facing development organisations [4]. In the case of the MEP, perceptions around the need for the optometry program, professional infringement and scepticism around partner interests were surprising findings in a context of a burden of visual impairment and lack of spectacle coverage in Mozambique [16]. This can be attributed to a lack of understanding of the profession of optometry and the socio-economic consequences of visual impairment. Therefore, advocacy for new programs is essential to the understanding and acceptance of donor-driven training programs in targeted countries. The MECC proved a key platform through which the MEP was able to influence policy and drive the optometry agenda in Mozambique. Local engagement through this strategic alliance therefore proved valuable in terms of the eventual acceptance of the profession of optometry.

Furthermore, demonstrating the potential value of the training program through large-scale launch initiatives may create a positive association between the program and its intended value. This can be supported by having well recognised and respected local personalities or leaders launch the program, which may drive broader public support for the project.

The finding that there was a lack of clarity between the project component and the longer-term optometry program of the funding initiative is not unique to the MEP. Many people use the terms ‘project’ and ‘program’ interchangeably, yet there is a distinct difference between the two terms. A project typically relates to a set of specific activities within a defined timeframe, while a program has a broader scope, potentially consisting of several ongoing projects within an extended timeframe [7]. Weaver noted that these two terms have long been confused or used interchangeably on many major projects [29]. This creates a lot of confusion in long term projects which are initiated by donor funds and then handed over to local ownership after a fixed period of time. Therefore, clearly delineating the donor-funded project component from the longer term, locally-owned professional program and its requirements is important for mutual understanding. However, there must be a link between the project activities and overall program.

The MEP embarked on a strategic public relations campaign, and in the process, built a strong brand as evidenced by their global support on social media platforms such as Twitter and Facebook. Publicity activities included a professionally designed and regularly updated website, production and dissemination of mini-documentaries, regular social media updates, as well as strong representation at key professional conferences globally. Through these activities the MEP established a reputable brand within the eye care community globally.

The MEP benefited from this strong international publicity campaign despite criticisms that these did not extend locally to the extent they may have been required. Recommendations from the Irish Aid evaluation report indicated the need for local public awareness tools targeting different segments of the population and institutions. It further recommended that public dissemination at institutional level should be strong in order to improve awareness among other health professionals regarding the role and responsibilities of optometrists as part of the eye care team [13].

**Fig. 2:**
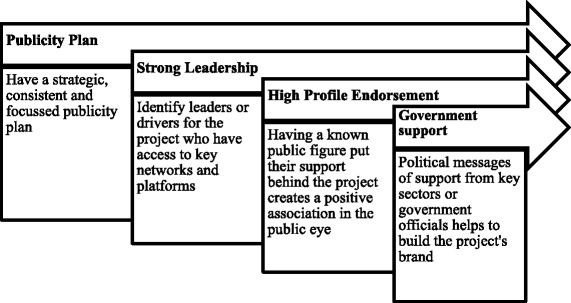
Factors supporting the development of a project’s brand

An analysis of factors positively contributing to the MEP’s public image included (Fig. 2):

- Having a publicity plan;
- Strong leadership;
- Having high profile endorsement, and
- Government support.

Partnership with local non-governmental organisations was also noted as positively reinforcing the project’s identity and brand.

Central to a program’s sustainability is the recognition of the proposed profession by relevant government authorities. This marker for the MEP was the employment of first optometry graduates by the MoH.

Literature cites several necessary components characterising the official recognition of professions. These include [21]:

- The existence of a legal recognition for work in the profession;
- The existence of a well-based and developing theoretical and practical body of knowledge informing the professional training and development; and
- The existence of a professional association with a code of ethics and the authority to make decisions for the profession.

Development projects tend to focus on implementing the project’s activities and meeting funder outputs. However, inherent in the sustainability of development investments is funders’ obligation to develop the system to support the program. The absence of professional guidelines may therefore result in challenges around the placement of optometrists within the health system. Any new cadres developed must form part of an established structure within the health system. If the structure does not support the model or exit competencies of the training program, then the model will fail as it is not supported by the system [19].

While optometry has been included in the broader eye health plan for Mozambique 2011-2016, not having a regulatory framework in place may result in unqualified persons opening or owning optometric practices since the general public will not know how to differentiate between those with and without proper qualifications [21]. Furthermore, authorities will have no means by which to accredit new training programs. The benefits of establishing a regulatory framework for the new profession are highlighted in Table 2 below.

**Table 2:** Value of a professional regulatory framework

Regulatory Framework Benefits	
Defines the role of practitioners within the healthcare context	
Allows for licensing and government oversight of training	
Defines responsibilities ad accountabilities of practitioners	
Informs inter professional collaborative practice relations	
Promotes public interest and risk protection	
Drives continuing professional development	

The MEP, through its research agenda has, however, developed a competency framework for optometrists in Mozambique [24]. This framework can therefore form the basis of any regulatory developments in the country.

Professional rivalry is a known concept amongst professions in the same field. Research has shown that ill-informed perceptions around the construction of other professions’ roles often results in views that are dissonant with those professions’ constructions of themselves [15]. This reflected professional rivalry is often based on misunderstanding, which is in keeping with the findings of this research. Advocacy efforts are therefore important in driving understanding of, and support for, new professions. Therefore in the case of optometry, in addition to providing an official legal basis for recognition of this new profession, advocacy efforts should also be aimed at contextualising the role of optometrists within both the health and eye care teams. This can enhance efforts towards broader local recognition and professional acceptance.

It is rare for refractive care practitioners to be distributed throughout a country in a way that ensures equitable access for all communities. Generally, the poorer and more rural a community is, the more limited access to refractive care will be [8]. This was confirmed by research in Mozambique which highlighted access to refractive services by rural communities as a significant barrier [26]. Despite human resource development models and selection practices aimed at encouraging rural practice amongst health care workers, research has shown that there have been limited successes in this regard [30]. It is therefore necessary to recognise constraints to graduate deployment and consider various strategies to lure professionals to remote practice settings which may include bursar-linked placements, compulsory community service, rural allowances or internships in public health facilities as applied in countries such as South Africa.

The MEP evaluation team acknowledged the risk that new graduates may be attracted by the private sector in major cities if government does not reached a state of readiness to absorb them. This requires that the MoH create funded posts in the public health sector, as well as provide adequately equipped facilities in order to attract and retain new professionals within the public health system. As would be expected by an emerging economy, there is high demand for health science graduates by the private sector in Mozambique which serves as direct competition for the MoH in the recruitment of new graduates. In the absence of a regulatory framework, it becomes difficult to control where, how or by whom graduates are employed. It is therefore plausible for development projects in human resources for health to consider a mixed model placement strategy which will likely be an inevitable outcome, since a growing private sector also promotes economic and social development; ultimately the intention of development projects.

Eye care has historically not been high on the agenda of health care in developing countries, with many facing more serious health care challenges such as HIV and Tuberculosis. Consequently, more attention should be directed towards policies and strategies which address URE in these countries. The MoH in Mozambique recognised this by including Optometry into its Five-Year Strategic Plan Vision 2020, a consequence of the MEP’s advocacy efforts through the MECC.

It is argued that while most higher education programs are a product of national developments and policies, the influence of globalisation on the internationalisation of professions cannot be ignored [28]. In the absence of a regulatory framework, professional identity may therefore be shaped by multiple factors, including the country’s frame of reference, regional alliances and burgeoning commercial interests. Research in other professional fields indicates a shift in regulatory logic where historical efforts to separate commercial interests from professional practice were previously suppressed, but are now being embraced. In order for developing countries to meet their objective of serving the neediest, and funders to receive a good long term return on their investment, the risks of graduates leaving en masse to an emerging private sector need to be addressed via regulation [25].

Insufficient capacity and resources have been identified as some of the challenges to professional regulation within the context of healthcare, even in developed countries [5]. The Department of Health in the United Kingdom, in its review of professional regulation legislation recommended proactivity rather than reactivity in designing regulatory models. However, it noted the challenges changing culture and relationships in health care, as would be the case with the introduction of a new profession into the health system, and the difficulties legislating for these. However, it remains a necessity, despite the length of time it may take to enforce legislation and promote self-determination of professions.

**Fig. 3:**
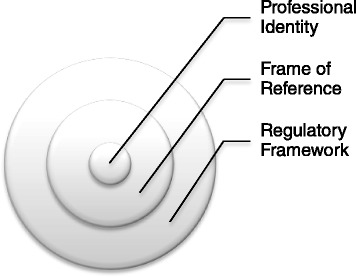
Regulatory Frameworks provides a frame of reference for professional identity

Therefore, as part of the development objective of capacity building, projects must prioritise a draft regulatory framework which can provide a frame of reference, help promote professional identity and inclusion, as well as guide the profession towards official recognition for its longer term development (Fig. 3).

Countries can learn from other examples through their development partners. Working examples of regulatory frameworks may be readily shared and adapted for the local context and form a guide which can be used by professional activists from the first cohort who can drive the future shaping of the profession. Therefore, promoting the establishment of an association of student and graduate optometrists in a new country of implementation is an important and necessary milestone for the future development of professional optometry in-country.

While not a consideration in the term of the project, the need for continuous professional development and postgraduate training is also an important next step in the ongoing development of the profession of optometry in Mozambique. Promoting alumni platforms and events to maintain and develop relationships with graduates will further serve to reinforce professional identity.

## Conclusion

The challenge of developing new professional cadres within the context of meeting developmental objectives is complex and noted. However, strategic advocacy around necessary professional development activities early in the project have the potential to influence long term health and human resource policy in the country. Establishing a professional regulatory framework within the local health system should therefore form a central component of every development project designed to train new health professions.

Local human resource and regulatory environments will always contribute uniqueness to each country’s professional guidelines. In the context of globalisation, however, there are many examples development organisations can draw from. Organisations are hereby encouraged to pursue a draft framework concurrent to the establishment of new optometry programs. Graduates and future leaders within the profession then have a working guide which can be used to shape professional practice within these countries, taking into account local dynamics. Frameworks should support evolving professional practice in a changing healthcare environment.

Therefore the recommendations from this research to development project planners include clearly delineating the shorter term project and its related activities as contributing to the establishment of the professional training program; identifying and addressing professional identity challenges and factors contributing to these; explicitly positioning optometrists within the local eye health team by identifying scope of practice and competencies in relation to related professions; and the prioritisation of advocacy efforts around local professional recognition through established, respected structures.

Efforts towards official recognition may begin as a local student council with representation from the MoH, which can then evolve into a professional regulatory council, guided by the local need and experience. Having good relations with politicians therefore serves to drive this agenda so that the semblance of a structure is in place for regulatory control at the point of graduation of the first professionals.

Official recognition processes are not simple and take time. However, project implementation and the development of initial professional regulatory structures can and must happen concurrently so that graduate professionals and their qualifications are legitimised, and the longer term future of the profession is guarded.

### Ethics approval and consent to participate

Research was approved by the Dublin Institute of Technology’s Research Ethics Committee and verbal consent to participate was received from all participants.

### Consent for publication

Not applicable.

### Availability of data and materials

The dataset will not be shared, owing to the confidentiality clause in the consent process, where participants were advised that the data will be securely stored and only accessible to the researcher. The raw data from which underlying conclusions in this paper were drawn is kept in secure storage by the researcher who is based in South Africa, in password protected computers and controlled access hard copy.

## Abbreviations

BHVI: Brien Holden Vision Institute
DIT: Dublin Institute of Technology
MECC: Mozambique Eyecare Coalition
MEP: Mozambique Eyecare Project
MoH: Ministry of Health
URE: Uncorrected refractive error
WHO: World Health Organization
